# Chronic cOronary Syndrome in Swedish PRImary care (COSPRI)—a study protocol for a 5-year cluster randomized controlled trial on a novel package versus standard investigation in patients with suspected chronic coronary syndrome referred from primary health care

**DOI:** 10.1186/s13063-025-08911-w

**Published:** 2025-06-21

**Authors:** Staffan Nilsson, Fade Gabro, Erik Stertman, Lars Bernfort, Mats Fredrikson, Pontus Henriksson, Peter Johansson, Lisa Kastbom, Anita Kärner Köhler, Johan Lööf, Ghassan Mourad, Eva Olsson, Carlos Valladares, Carl Johan Östgren, Sofia Sederholm Lawesson, Jan Engvall, Fredrik Iredahl

**Affiliations:** 1 Department of Health, Medicine and Caring Sciences, Primary Health Care Center, Linköping University, Linköping, Sweden; 2 Department of Health, Medicine and Caring Sciences, Linköping University, Linköping, Sweden; 3 Department of Biomedical and Clinical Sciences and Forum Östergötland, Linköping, Sweden; 4 Department of Biomedical and Clinical Sciences, Linköping University, Linköping, Sweden; 5 Center for Medical Image Science and Visualization, Linköping, Sweden; 6 Department of Cardiology in Linköping, and, Department of Health, Medicine and Caring Sciences , Linköping University, Linköping, Sweden; 7 Department of Clinical Physiology, and, Department of Health, Medicine and Caring Sciences , Linköping University, Linköping, Sweden; 8 Wallenberg Center for Molecular Medicine, Linköping University, Linköping, Sweden

**Keywords:** Coronary artery disease, Chronic coronary syndrome, Primary health care, Exercise test, Single photon emission computed tomography (SPECT)

## Abstract

**Background:**

This trial aims to assess the effectiveness of a novel diagnostic package in the investigation of symptomatic chronic coronary artery disease (CAD), with a focus on reducing the time to diagnosis and improving risk assessment, compared to the current standard investigation approach. The package investigation is comprised of combined single photon emission computed tomography (SPECT), echocardiography, coronary artery calcification (CAC) scoring, not previously evaluated in Sweden for individual cardiovascular prevention, and CadScore, an acoustic risk score. The standard investigation is comprised of exercise bicycle stress testing and echocardiography.

**Methods:**

The trial involves patients with a pre-test probability > 15% for significant CAD referred from primary healthcare centers (PHCs) in Region Östergötland (population 471,241 in March 2023) in south-east Sweden. All the 47 PHCs of the region will be invited to participate and will, after approval, be cluster randomized into two groups: package investigation versus standard investigation. The primary outcome is a composite measure comprised of waiting time to invasive coronary angiography or communication of non-invasive myocardial ischemia investigation results. Secondary outcomes include major adverse cardiovascular events (MACE), cost per patient, signs of reversible ischemia on any test, radiation exposure, and adherence to prescribed cardioprotective drugs.

**Discussion:**

This trial addresses the urgent problem of chest pain and dyspnea assessment in primary care and aims to speed up diagnosis, reduce the need for specialist consultations, and potentially improve patient outcomes, with referral to SPECT directly from PHC in comparison with the widely used exercise test. The novel approach includes CAC scoring. Additionally, the utility of acoustic CadScore in reclassifying the risk of CAD is explored.

Trial registration.

The trial was registered on March 11, 2023, at ClinicalTrials.gov with the identifier: NCT05782582.

## Administrative information

 Note: the numbers in curly brackets in this protocol refer to SPIRIT checklist item numbers. The order of the items has been modified to group similar

**Table:** 

Title {1}	Chronic cOronary Syndrome in Swedish PRImary care (COSPRI)—a study protocol for a 5-year cluster randomized controlled trial on a novel package versus standard investigation in patients with suspected chronic coronary syndrome referred from primary health care
Trial registration {2a and 2b}.	Identifier: NCT05782582 at ClinicalTrials.gov 2b N/A To our knowledge, ClinicalTrials.gov does not collect all items from the World Health Organization Trial Registration Data Set.
Protocol version {3}	Issue 1.1
Funding {4}	The trial receives grants from Swedish Research Council (grant number 2023–05738) and ALF Grants, Region Östergötland (grant number RÖ−994,046, RÖ−999,705).
Author details {5a}	^1^ Primary Health Care Center, Department of Health, Medicine and Caring Sciences, Linköping University, Linköping, Sweden ^2^ Department of Health, Medicine and Caring Sciences, Linköping University, Linköping, Sweden. ^3^ Department of Biomedical and Clinical Sciences and Forum Östergötland ^4^ Department of Biomedical and Clinical Sciences, Linköping University, Linköping, Sweden. ^5^ Center for Medical Image Science and Visualization ^6^ Department of Cardiology in Linköping, and Department of Health, Medicine and Caring Sciences, Linköping University, Linköping, Sweden ^7^ Department of Clinical Physiology, and Department of Health, Medicine and Caring Sciences, Linköping University, Linköping, Sweden
Name and contact information for the trial sponsor {5b}	Region Östergötland Email:region@regionostergotland.se
Role of sponsor {5c}	The sponsor and funders have had no role in the design of the study and have no role in the collection, analysis, interpretation of data or in writing the manuscript.

## Introduction

### Background and rationale {6a}

Chest pain, a main symptom of coronary artery disease (CAD), is common in primary care and is often triggered by benign causes (1, 2). Nevertheless, primary care physicians may need to consult a cardiologist in about one of four cases of non-acute chest pain (3). In addition, dyspnea, another common symptom in primary care, may be triggered by CAD, but has a higher risk of all-cause mortality (4). CAD causes premature death and is one of the main causes of loss of healthy life years due to disability in industrialized countries (5). The annual cost of CAD in the European Union is estimated at €19,248 million, accounting for 1.29% of the total health care expenditure (6). Notably, only 7% of patients with suspected CAD referred for assessment exhibit significant coronary stenosis suitable for revascularization (7).

In the investigation of suspected CAD, adopting a structured approach is necessary to effectively utilize resources. In 2019, the European Society of Cardiology (ESC) published guidelines on chronic coronary syndromes, advocating for an approach based on the assessment of pre-test probability (PTP) for significant coronary stenosis (8). PTP, as per ESC guidelines, is estimated on the patient’s age, sex, and symptom characteristics (8, 9). The PTP for significant coronary stenosis, given a specific combination of age, sex, and symptoms, is considerably lower than in the previous guidelines issued in 2013 (9). In 2024, after the study start, ESC published updated guidelines with risk assessment based on Risk-factor-weighted clinical likelihood (10).

According to ESC 2019 guidelines, in the presence of any traditional cardiovascular risk factor e.g., family history of premature CAD, smoking, dyslipidemia, hypertension, diabetes mellitus, symptomatic patients with intermediate risk of significant CAD (PTP > 15%) are recommended to undergo a functional imaging-based technique, such as SPECT, despite the greater accessibility of exercise stress testing (8). The rationale for this recommendation is that sensitivity to detect anatomically and functionally significant CAD is 87% (95% CI 83–90) for SPECT and 58% (95% CI 46–69) for exercise stress tests with a specificity of 70% (95% CI 63–76) and 62% (95% CI 54–69), respectively (11). Additionally, an echocardiogram at rest is recommended in the basic investigation and may reveal indirect signs of chronic CAD, valvular heart disease, heart failure, or cardiomyopathies (12). Another modality, easy-to-use and potentially suitable in a primary care setting to rule out CAD, is the acoustic CadScore (13).

To our knowledge, coronary artery calcification (CAC) scoring has not yet been evaluated in Sweden as a tool in individual cardiovascular prevention. The CAC score has been shown to predict future cardiovascular events, independently of age, sex, and ethnicity, and could lead to reclassification of cardiovascular risk scores based on age, sex, and traditional risk factors (14). According to European and US guidelines, CAC scoring can be used as a supplement to traditional risk factors when determining cardiovascular preventive treatments and facilitating informed choices (8, 15). Performing a computed tomography (CT) scan of the heart to calculate the CAC score is a straightforward procedure that adds only a small amount of radiation, < 0.4 millisievert (mSv).

Rapid and conclusive investigations should be valuable for patients. Therefore, within the COSPRI (Chronic cOronary Syndrome in Swedish PRImary care) trial, we have combined single-photon emission computed tomography (SPECT), echocardiography, and CAC scoring into a diagnostic package, in most cases administered on one single day. The package testing approach is potentially more time efficient, since a step-wise approach demands repeated decision making by the health care provider. The package testing approach also includes additional diagnostic information that potentially could lead to primary and secondary preventive treatment decisions.

### Objectives {7}

We hypothesize that the diagnostic package will shorten the time to conclusive results, including the rule out of CAD, time to diagnosis, and invasive coronary angiography. We further hypothesize that the use of the diagnostic package will improve conditions for cardiovascular prevention, streamline clinical decision-making for primary care physicians, reduce the number of diagnostic tests at different time points, and reduce the necessity for cardiology consultations.

### Trial design {8}

**Fig. 1:**
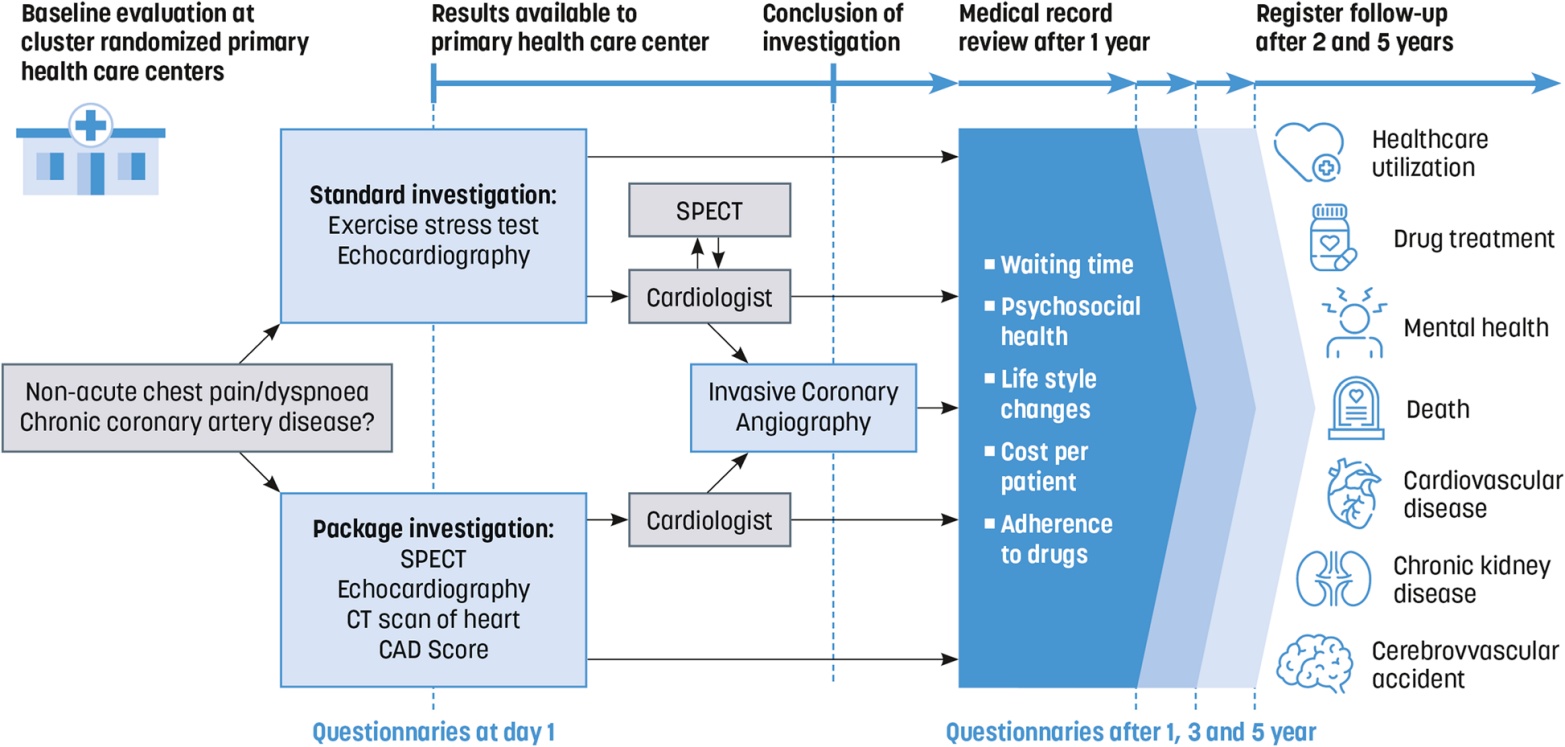
Flow chart of the Chronic cOronary Syndrome in Swedish PRImary care (COSPRI) trial

COSPRI is a cluster-randomized trial with parallel groups. A cluster is defined as a PHC. Invitations to participate will be extended to all 47 PHCs in Region Östergötland. Those PHCs consenting to participate will be randomly assigned to either the investigation package or to the existing diagnostic routines. Before randomization, the PHCs will be stratified as “large” or “small” depending on their number of listed individuals in order to ensure that there will be approximately the same number of patients in the two groups. To ensure uniformity across all participating units, standardized start-up training and trial protocol information will be provided in both trial arms. Figure 1 provides a schematic overview of the trial.

## Methods: participants, interventions, and outcomes

### Study setting {9}

This trial includes patients consulting in primary health care who, by the referring primary care physicians, are assessed to have a PTP for the presence of a significant coronary stenosis exceeding 15%. The study setting was designed not to interfere with normal clinical referral routines. Region Östergötland, in southeast Sweden, has 47 primary health care centers (PHCs) and 471,241 inhabitants as of March 2023. There are two Physiology Clinics in the region, both participating in the study, one at a university hospital and one at a regional hospital. The package investigations are all carried out at the university hospital, and the standard investigations are all conducted at either the university or regional hospital. In addition, standard investigations can be carried out by a private cardiology consultant, situated in the same city as the university hospital.

### Eligibility criteria {10}

#### Cluster level

All PHCs within Region Östergötland, publicly or privately run, are eligible to participate in COSPRI. Since the study demands some effort by the physicians at the PHC, explicit willingness to participate is mandatory.

#### Individual level

Inclusion criterion: Symptoms judged to be compatible with PTP > 15% for symptomatic chronic CAD.

Exclusion criteria are shown in Table 1.

**Table 1:** Exclusion criteria in the COSPRI-study

Exclusion criteria
Suspicion of acute coronary syndrome at medical contact
Previously diagnosed acute myocardial infarction (MI)
Previous revascularization with percutaneous coronary intervention (PCI) and/or coronary artery bypass grafting (CABG)
Previously proven ischemia exceeding 10% left ventricular mass on myocardial single-photon emission computed tomography (SPECT)
Left Bundle Branch Block on resting electrocardiogram (ECG)
Ventricular pacing rhythm on resting ECG
Age below 18 years
People whose meaning, due to illness, mental disorder, weakened state of health or any other similar condition cannot be obtained, to be included in this research project
Insufficient understanding of spoken and written Swedish language

### Who will take informed consent? {26a}

Patients receive written information along with scheduling details from one of the two Physiology Clinics or the privately run cardiology consultant. Written informed consent will be obtained by a trained research nurse on the day of the investigation. No economic compensation will be provided to trial participants, and all trial participants have the option to withdraw from the trial at any time.

### Additional consent provisions for collection and use of participant data and biological specimens {26b}

No additional consents are planned.

### Interventions

#### Explanation for the choice of comparators {6b}

We will evaluate if the package investigation reduces the time for ruling in or out myocardial ischemia in comparison with the standard investigation.

### Intervention description {11a}

#### Intervention arm

Trial participants will undergo a package investigation comprised of:

1. Resting ECG
2. Risk evaluation based on PTP
3. Echocardiography
4. Exercise bicycle stress test (converted to pharmacological provocation if target heart rate is not reached or if treated with high dose beta-blockage, preventing target heart rate, or if the trial participants are not able to ride a bicycle for any reason) with injection of isotope for SPECT
5. Scanning for myocardial perfusion
6. CAC scoring with cardiac CT scan
7. Registration for CadScore, an acoustic risk score calculated on PTP and wavelet analysis of heart sounds

Numbers 1–7 will be completed within 1 week. If signs of myocardial ischemia are revealed under stress conditions at no. 5, a follow-up scan at rest will be performed 2 days later.

#### Comparator arm

Trial participants will undergo standard investigation comprised of:

1. Resting ECG
2. Risk evaluation based on PTP
3. Echocardiography
4. Exercise bicycle stress test

Number 3 and 4 will, in most cases, not be performed on the same day. The order and the waiting time between the investigations are decided due to clinical prioritization at the performing Physiology Clinic or at the privately run cardiology consultant.

### Criteria for discontinuing or modifying allocated interventions {11b}

The study participants can withdraw from the study at any time. In both study arms, the treating physician is free to add investigations and medical treatment, according to own deemed necessary.

### Strategies to improve adherence to interventions {11c}

A trained study nurse will distribute study questionnaires shortly after study inclusion and again after 1, 3, and 5 years by e-mail or regular mail. If there are no answers received, the nurse will contact the participant for support and assistance.

### Relevant concomitant care permitted or prohibited during the trial {11d}

Besides the different investigations within the two trial arms, participation in the trial has no impact on care or investigations that trial participants receive through routine healthcare procedures.

### Provisions for post-trial care {30}

Normal patient injury insurance applies. Compensation for any lost income, travel, or.

other expenses will not be paid.

### Outcomes {12}

Primary outcome measure.

**Fig. 2:**
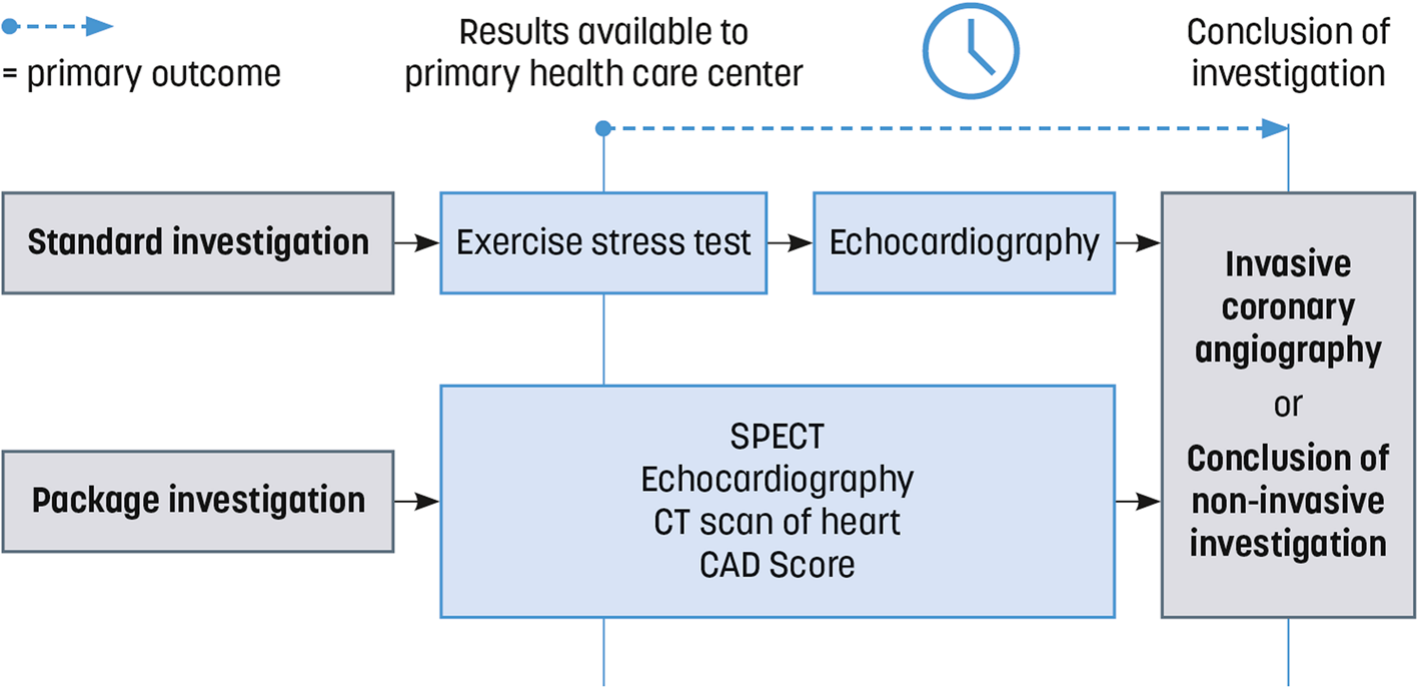
Descriptive figure of the primary outcome

The waiting time, counted in days, begins on the date when the results of the package investigation (package investigation group) or exercise bicycle stress test (standard investigation group) are approved and immediately available for the physician at the PHCs. It extends until the day of performance of invasive coronary angiography or until the communication of results, from the completed non-invasive myocardial ischemia investigation to the trial participant (patient), e.g., by letter, physical contact, telephone, or video call, as reported in medical records (Fig. 2). In the absence of follow-up contact in medical records, the date of the last cardiac investigation will be used (16). It is important to note that waiting time for the initial investigation will not be included in this measurement. Waiting time from primary health care referral to the initiation of standard or package investigation will also be measured but not included in the primary outcome measure.

Secondary outcome measures (Fig. 1).

Complication to disease:

- Major adverse cardiovascular events (MACE), a composite endpoint including cardiovascular death, in-hospital care for acute non-ST-elevation myocardial infarction (NSTEMI) or ST-elevation myocardial infarction (STEMI), emergency coronary revascularization, or stroke, will be obtained from the SWEDEHEART register, the National Patient Register, and the National Cause of Death Register (17–19).
- Presence of pathological Q-waves on resting ECG.
- Signs of reversible ischemia on any test.
- Radiation exposure per participant measured in mSv.

Risk factors of chronic coronary artery disease and their management:

- Patients’ adherence to prescribed cardioprotective drugs (20, 21). Calculations will be based on data from the National Prescribed Drug Register on prescribed and dispensed cardioprotective drugs (22).
- Smoking.
- Alcohol consumption measured by the Alcohol Use Disorders Identification Test (AUDIT). Score range: 0–40. (WHO publications 2001) (23).
- Coffee consumption will be measured with questions about how often and how many cups of coffee are consumed.
- Physical activity will be measured by two categorical questions asking for the level of physical activity (24).
- Physical fitness will be measured by the International Fitness Scale (IFIS) (25).
- Dietary habits will be measured by five categorical questions about consumption of fruit, vegetables, snacks, and soft drinks during the last week (26).
- Heart-focused anxiety will be measured by the Cardiac Anxiety Questionnaire (CAQ) (27).
- Generalized anxiety will be measured by the Brief Measure for Assessing Generalized Anxiety Disorder (GAD-7) (28).
- Symptoms of depression will be measured by the Patient Health Questionnaire (PHQ-9) (29).
- Sleep quality will be measured by the Pittsburgh Sleep Quality Index (PSQI) (30).
- Dental health will be assessed using a 5-item questionnaire designed specifically for this trial.

Health economic analysis:

A health economic analysis of the costs and outcomes related to the procedures will be performed. Costs consist of healthcare resource utilization and trial participants’ loss of production, travel costs, and days on sick leave. The cost of downstream testing will be calculated based on the number of visits and contacts with primary care and hospital outpatient clinics and in-hospital care for relevant diagnoses, cardiac investigations, X-ray including coronary computed tomography angiography (CCTA), and magnetic resonance imaging (MRI) investigations of the upper part of the body (excluding head and extremities). Data will be collected by manual review of medical records or extracted from regional medical registries. Costs associated with trial participant loss of production and travel will be estimated from questions on being employed or not, working hours per week, and distance from civil registry address to the clinic of interest. Healthcare costs per participant will be calculated and compared between the package and the standard investigation groups. Health-related quality of life will serve as the outcome in the health economic analysis and will be measured by EQ-5D-5L and RAND-36 Swedish versions to estimate quality-adjusted life-years (QALYs) (31, 32). The incremental costs for package- vs. standard investigation will be divided by incremental QALYs for the same comparison to get the incremental cost-effectiveness ratio (ICER), i.e., cost per QALY, of package investigation vs. standard investigation.

### Participant timeline {13}

**Fig. 3:**
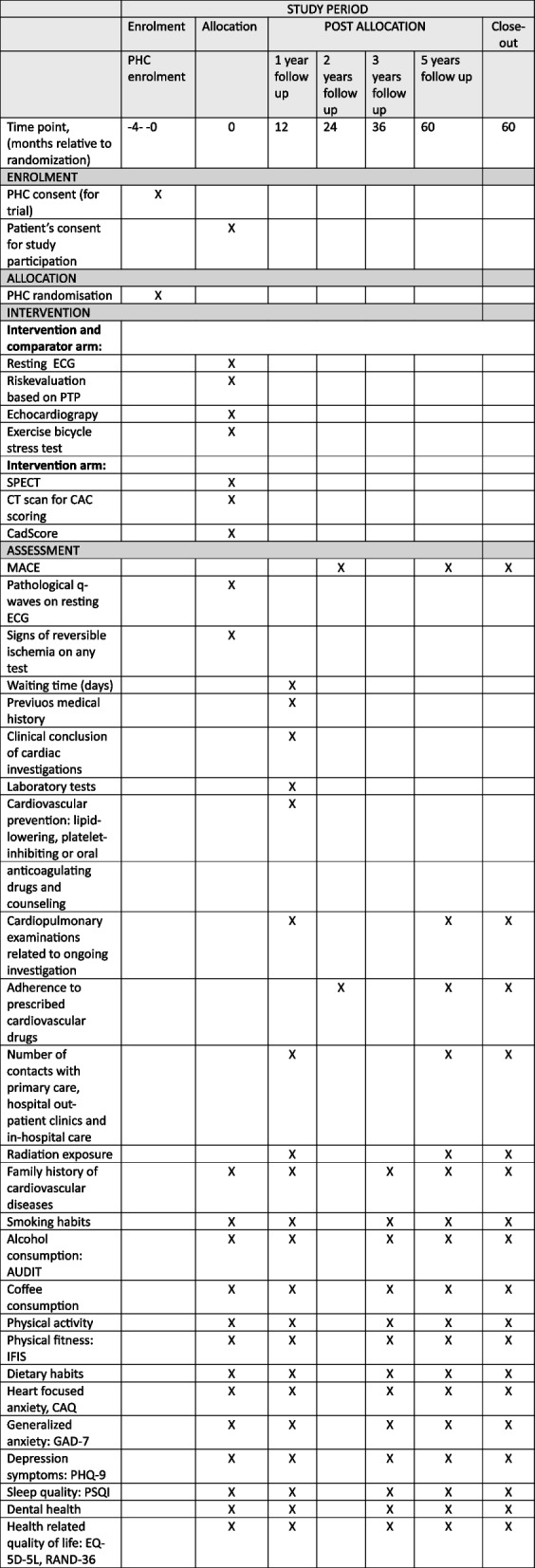
Schedule of enrollment, intervention and assessment in the Chronic cOronary Syndrome in Swedish PRImary care (COSPRI) study. PHC, Primary Healthcare Center; ECG, electrocardiogram; PTP, pre-test probability; SPECT, single-photon emission computed tomography; CT, computed tomography; CAC, coronary artery calcification; CadScore is an acoustic risk score; MACE, major adverse cardiovascular events; AUDIT, Alcohol Use Disorders Identification Test; IFIS, International Fitness Scale; CAQ, Cardiac Anxiety Questionnaire; GAD-7, Generalized Anxiety Disorder; PHQ-9, Patient Health Questionnaire; PSQI, Pittsburgh Sleep Quality Index; EQ-5D-5L, European Quality of Life 5 Dimensions 5 Level Version

Trial participant timeline on the individual level is demonstrated in Fig. 3.

### Sample size {14}

The sample size calculation is based on a comparison of the two mean waiting time (days) values, based on historical data, in a cluster randomized design where the clusters are the PHCs. We have used 18 clusters for the calculation, and we estimate the within-cluster correlation to be 0.1. Since the size of the clusters differs in size, we included a coefficient of variation of 0.46, based on the actual sizes of the PHCs. We have assumed the mean number of waiting time to be 37 days in the standard investigation group and 22 days in the package group, with a standard deviation of 39 and 16, respectively. This assumption is based on historical data on waiting times and a regional dialog between representatives from Primary health care, Clinical Physiology, and Cardiology Departments prior to the start of the study. With these assumptions, we estimate, with a power of 80% and a significance level set to 5%, that we need 416 participants, 208 per group. We expect that there will be some dropouts, so we increase the number of participants to 250 in each group.

Interpretation of possible outcomes: A statistically significant difference between groups means that there is more than a 15-day mean difference in waiting time for invasive coronary angiography or information on results of the non-invasive myocardial ischemia investigation. A non-significant difference between groups indicates that the package investigation does not save more than 15 days in comparison with the standard investigation. The 15-day difference is judged by the regional dialog, in this intermediate to high-risk group, to be clinically relevant.

### Recruitment {15}

Cluster level: Region Östergötland has 47 PHCs and 471,241 inhabitants as of March 2023. Invitations to participate will be extended to all 47 PHCs. Those consenting to participate will sign a study agreement. All 47 PHCs will be randomly assigned to one of the two study groups, and those consenting will be informed about their group affiliation. The PHCs not consenting may be added to the study later if deemed necessary to achieve the desired number of participants and if they are then willing to participate.

To ensure uniformity across all participating units, standardized start-up training and trial protocol information will be provided in both trial arms.

Individual level: To keep physicians and staff at all participating PHCs updated, COSPRI newsletters will be sent on a regular basis.

### Assignment of interventions: allocation

#### Sequence generation {16a}

Not applicable due to cluster randomization.

### Concealment mechanism {16b}

Not applicable due to cluster randomization.

### Implementation {16c}

Not applicable due to cluster randomization.

## Assignment of interventions: Blinding

### Who will be blinded {17a}

Neither trial participants, primary care physicians, nor researchers will be blinded to assignment to package or standard investigation.

### Procedure for unblinding if needed {17b}

Not applicable due to unblinded assignment.

### Data collection and management

#### Plans for assessment and collection of outcomes {18a}

##### Baseline data

Information on fulfillment of inclusion criteria and absence of exclusion criteria, age, date of birth, and PTP as assessed by the primary care physician are collected on day 1 (Fig. 1). Information on whether participants are working, the number of work hours per week, smoking habits, exposure to passive smoking, family history of heart infarction, angina pectoris, or stroke among parents, siblings, or children (occurring before age 65 for women and 55 for men), and, if applicable, the age at which menstruation ceased, will be collected via questionnaires distributed shortly after day 1 (Fig. 1). Information on participants’ previous medical history, including occurrences of stroke, transient ischemic attack, or intervention with weight-reducing gastric bypass operation of any kind, along with the year of first registration, and assessed Risk-factor-weighted clinical likelihood according to ESC 2024 guidelines, will be collected by reviewing medical records after 1 year (Fig. 3). Additionally, existing diagnoses and the year they were first diagnosed, such as essential hypertension, chronic kidney disease, diabetes mellitus type 1 or type 2, familial hypercholesterolemia, diaphragmatic hernia or esophagitis, migraine, breast, colon, or prostate cancer, anxiety, depression, psychotic illness, sleep disorder, chronic obstructive pulmonary disease (COPD), chronic inflammatory disease of the musculoskeletal system, psoriasis, or a diagnosis of post-COVID symptoms lasting at least 2 months, will also be collected via review of medical records after 1 year (Figs. 1 and 3).

#### Trial data

Pre-test probability.

**Fig. 4:**
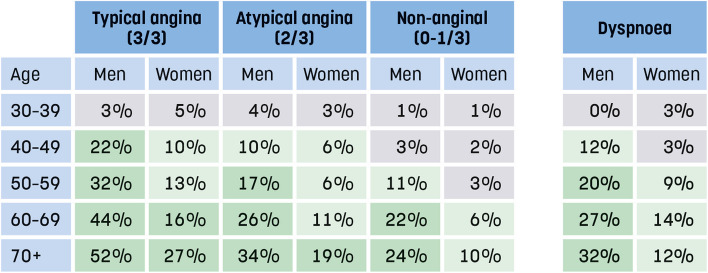
Pre-test probability (PTP) for obstructive coronary artery disease. Constricting discomfort in chest-jaw region and/or arms (1p), triggered by exertion (1p), relieved by rest or short-acting nitrates (1p). Typical angina 3/3, atypical angina 2/3, non-anginal 0–1/3. Modified from ESC Guidelines for the diagnosis and management of chronic coronary syndromes 2019

At referral in PHCs, estimation of the PTP is mandatory on the referral form for the package investigation and for the exercise bicycle stress test (standard investigation group). A table derived from current ESC guidelines is available adjacent to the referral form (Fig. 4) (8). If discomfort, pain, or pressure in the chest or any other angina equivalent localization is present, the remitter is recommended to use the PTP table for typical, atypical, or non-anginal symptoms. If dyspnea is the dominating symptom, with pain, discomfort, or pressure never or seldom present, the remitter is recommended to use the PTP table for dyspnea (Fig. 4). After ticking a box, a drop-down list appears prompting a mandatory choice of a precise PTP figure, i.e., from 0 to 52%, derived from either of the two tables shown in Fig. 4.

The following trial data will be collected by review of medical records about 1 year after day 1.

Clinical outcome.

The conclusion of the cardiac investigation will be sought in medical records. If the investigations show signs of chronic CAD or some other heart disease, one or more of the following will be noted: clinically assessed angina pectoris, significant coronary artery stenosis, heart failure with ejection fraction < 50% or > 50%, clinically significant heart valve disease, or suspected or proven cardiomyopathy. In addition, any other cardiac disease will be noted with a mandatory free-text description. If no signs of cardiac disease are found, the cause of chest pain or dyspnea will be noted as musculoskeletal, gastroesophageal reflux, anxiety, depression, COPD, reduced physical fitness, obesity, no information given, or any other cause, with the possibility for a free-text description.

Laboratory tests.

No blood samples will be drawn exclusively for the trial. All blood tests will be performed by the Department of Clinical Chemistry or its point-of-care operations at the participating PHCs. This laboratory is accredited according to ISO-IEC 15189, which means that the quality of analyses and the reporting of results is closely monitored according to the standard. The blood tests that will be collected by reviewing medical records, if available, after 1 year are hemoglobin (g/L), creatinine (mmol/L), total cholesterol (mmol/L), low density lipoprotein (LDL) cholesterol (mmol/L), hemoglobin A1C (HbA1C) (mmol/mol), and N-terminal pro-B-type natriuretic peptide (NT-proBNP) (ng/L).

Cardiovascular prevention.

Ongoing prescription of lipid-lowering, platelet-inhibiting, or oral anticoagulating medication and, if so, the specific drug will be noted. If counseling was documented regarding physical activity, smoking cessation, dietary habits, or mental stress, it will be noted.

Cardiopulmonary examinations related to ongoing investigation.

Occurrence of abnormal cardiac (and/or pulmonary) status, and if so, the descriptive text is copied and pasted from the medical record, in the digital case report form.

Performance date and results of CCTA, 24-h ECG, comprehensive spirometry at the physiology clinic, dynamic spirometry at the PHC will be collected from the medical records.

The date and results for SPECT, Dobutamine stress echocardiography, invasive coronary angiography will be noted by review of medical records after 1 year, and also collected from national or regional medical registries after 5 years (Fig. 3).

The type of and date for any radiological examinations related to the ongoing investigation, such as X-ray, CT, or MRI, of the upper part of the body excluding the head or upper extremities will be noted. The results will be noted as normal, difficult to assess, or clearly pathological.

Number of days on sick leave from day one will be collected by review of medical records after 1 year and by data retrieval from Statistics Sweden after 5 years.

Radiation exposure.

Data on radiation exposure will be collected by regional or national registries after 1 and 5 years.

CadScore data.

For participants in the package group CadScore calculation, information about age, sex, and symptoms will be added to the audio recording, resulting in a composite probability of disease displayed on a scale of 0–100. The CadScore sum will not be given to the referring primary care physician but kept for strictly scientific analyses.

Risk factors of chronic coronary artery disease and their management.

Data will be collected by validated questionnaires (23–32). Smoking will be assessed by the question “Do you smoke?” with the response options (No, I have never smoked/No, I have quit/Yes, occasionally/Yes, daily) and quantified by the number of cigarettes smoked per day. Dental health will be assessed using a 5-item questionnaire designed specifically for this trial. Study questionnaires will be sent by digital routines or regular mail shortly after day 1, after 1, 3, and 5 years.

Major adverse cardiovascular events (MACE) and cardioprotective drugs.

Data will be retrieved from the National Patient Register, the Cause of Death Register (National Board of Health and Welfare), and SWEDEHEART (Uppsala Clinical Research Center, to calculate the number of MACEs. Additionally, data will be retrieved from the National Prescribed Drug Register (National Board of Health and Welfare) to track the prescription and purchase of cardioprotective drugs (22) (19) after 2 and 5 years (Fig. 3).

### Plans to promote participant retention and complete follow-up {18b}

Cluster level: Participating PHCs are contracted to stay within the study until the preset number of participants on the individual level is reached.

Individual level: Study participants will be contacted by e-mail or regular mail shortly after day 1 and after 1, 3, and 5 years by a trained study nurse in order to collect their answers to study questionnaires.

### Data management {19}

Participants in the trial will be assigned specific participant numbers for coding purposes. Pseudonymized medical record review and survey data will be entered in the secure web application Research Electronic Data Capture (RedCAP) and stored on a server at Linköping University (33, 34). All image material will be saved according to clinical protocols, and data for research purposes will be stored under pseudonymized conditions. Images will be stored pseudonymized digitally, ensuring that trial participants cannot be identified based on image materials. The audio recording itself in CadScore calculation will not be retained. Image and supplementary functional data, including blood pressure, pulse, pain response, grading of effort from the myocardial perfusion or control bicycle work test, and echocardiography will be stored in clinical databases within Region Östergötland. These data will then be exported through pseudonymization equipment to the research database at the Centre for Medical Imaging Science and Visualization (CMIV). The code keys will be stored on a separate digital medium by the principal investigator (PI) at Region Östergötland.

### Confidentiality {27}

Data will be pseudonymized and the RedCAP software ensures the secure data collection, storage, and maintenance both during and after the trial.

### Plans for collection, laboratory evaluation, and storage of biological specimens for genetic or molecular analysis in this trial/future use {33}

No biological specimens obtained during the conduct of the trial will be stored for future use.

### Statistical methods

#### Statistical methods for primary and secondary outcomes {20a}

Analyses will be performed according to the intention-to-treat principle. Conventional descriptive methods using means (with standard deviations) or medians (with interquartile ranges) for continuous data and proportions for categorical data will be used to compile data. Since the data are not totally independent due to the cluster design, we will use mixed models for the analysis. For the continuous outcomes (the primary hypothesis) we will use a linear mixed model, and for proportions (the secondary hypotheses), a logistic mixed model. The same model will be used to control for potential confounding.

### Interim analyses {21b}

No interim analyses will be conducted as we do not foresee any potentially serious outcomes.

### Methods for additional analyses (e.g., subgroup analyses) {20b}

In addition, analyses for each component of the package investigation (CAC-score, SPECT, and CadScore) are planned. Descriptive analyses will be performed to describe the components and investigate associations between the outcome and the components.

### Methods in analysis to handle protocol non-adherence and any statistical methods to handle missing data {20c}

Missing data for variables at random will be replaced by using multiple imputation. In case of systematic missing data, like if whole instruments (questionnaires) are missing, that will not be replaced.

*Plans to give access to the full protocol, participant level data*,* and statistical code {31c}.*

There are no plans for granting public access to the full protocol, participant-level data, and statistical code.

### Oversight and monitoring

#### Composition of the coordinating center and trial steering committee {5d}

The trial steering committee consists of an associate professor, a specialist in primary care FI, a principal investigator (PI) since 26th of March 2024, co-PI SN, also a specialist in primary care, JE, responsible for the ischemia investigations, SSL, a senior consultant cardiologist, and will be led by the PI.

### Composition of the data monitoring committee, its role and reporting structure {21a}

An independent data monitoring committee is not deemed necessary to ensure the quality of data and study endpoints. Forum Östergötland, a support function for clinical translational research at Region Östergötland and Linköping University, will conduct a start-up check of the trial. They will ensure that all documentation, including permits, agreements, and storage/archiving procedures at participating trial sites, is in place and good clinical practice will be followed when appropriate.

When the first 20 participants are included in the study, we will thoroughly analyze the inclusion procedure at the Physiology Clinics in Linköping and Norrköping, and at the privately run cardiac specialist clinic and collection of data from questionnaires answered by participants and noted in RedCAP. After the first year of the trial participant’s inclusion, an independent reviewer is planned to conduct a data review, comparing RedCAP data with source data from medical records as well as data collected at the two physiology clinics and a privately run cardiac specialist clinic participating in the trial. Towards the end of the time period for the inclusion of study participants, we are planning for an adjudication committee of independent researchers tasked with determining the prevalence of MACE in both study groups, i.e., package investigation or standard investigation, respectively.

### Adverse event reporting and harms {22}

Serious adverse event (SAE) is defined as clinically diagnosed myocardial infarction, deaths by any cause, or hospitalization for heart failure. Adverse event (AE) hospitalization for increased chest pain and indirectly diagnosed myocardial infarction, e.g., by pathologic q-wave. All members of the study staff at the participating Physiology clinics or privately run cardiologist consultants are encouraged to report any SAE or AE of which they become aware to the steering committee. SAE and AE apply only to individuals after given informed consent.

### Frequency and plans for auditing trial conduct {23}

Not applicable. There is no on-site auditing of the trial.

### Plans for communicating important protocol amendments to relevant parties (e.g., trial participants, ethical committees) {25}

The trial steering committee will communicate substantial protocol modifications (when applicable) to relevant parties (Ethical Review Authority), study staff, and study participants on the cluster level, i.e., PHCs.

### Dissemination plans {31a}

Results of the trial will be published in international peer-reviewed scientific journals and reported according to CONSORT 2010—extension to cluster randomized trials. There is no obligation to communicate the results to participants, neither on the cluster nor the individual level.

## Discussion

Chronic artery disease is a common disease that imposes significant public expenses and causes individual suffering. Although well-established methods for diagnosing symptomatic chronic CAD exist, their full potential is not being utilized, particularly in primary care where patients with symptoms suggestive of symptomatic chronic CAD are highly prevalent.

In the 2019 ESC guidelines on chronic coronary syndrome, in addition to PTP, the clinical likelihood is advocated as the basis for selecting non-invasive investigational modalities for significant CAD. However, the PTP estimation remains critically important, as it has been demonstrated that the annual risk of cardiovascular death or myocardial infarction is less than 1% when PTP is < 15% (7). Consequently, the ESC guidelines suggest that it is safe to defer further testing if PTP is < 15% in the absence of traditional cardiovascular risk factors. Moreover, testing when PTP is < 5% should only be conducted for compelling reasons (8). However, a knowledge gap exists regarding the suitability of this approach, as the risk assessment is primarily based on countries with a low risk of cardiovascular disease, while Swedish citizens are at a moderate risk. Using the waiting time from the day when results from package or standard investigations are available at the PHC (Fig. 2) until the conclusion of diagnostic assessment or until intervention by invasive coronary angiography as the primary outcome measure allows for comparison with other PHCs on a national and international basis. The alternative primary outcome of measuring MACE would not achieve sufficient statistical power within a reasonable time frame, also considering future possible changes of guidelines.

Since the initiation of the study start, the ESC has updated the recommended guidelines (10). This is compensated for by a 1-year post-investigation assessment of the risk-factor-weighted clinical likelihood.

Conventionally, primary care physicians follow a stepwise approach, ordering diagnostic tests sequentially, most often starting with exercise testing and referring to cardiologists if more advanced testing is considered needed, such as SPECT or CCTA. This approach may save resources but leads to delays in reaching a correct diagnosis. In this trial, we therefore have combined SPECT, echocardiography, and CAC scoring into a diagnostic package. This approach aims to determine whether patient complaints can be provoked by exercise, whether they are associated with the induction of ischemia, whether arterial calcification indicates prior coronary inflammation, and whether there are differential diagnoses evident at the echocardiographic examination. Additionally, prognostic information can be obtained to guide preventive pharmacological treatments. The acoustic CadScore calculation will be performed to assess whether it provides additional information in this population with a moderately increased risk. To not overwhelm the primary care physician with investigational results, the CadScore sum will not be given to the referring physician but be kept for strictly scientific analyses. This novel investigation package has not been studied previously and requires comparison with the current stepwise approach. Initially, the extensive package investigation may generate higher healthcare costs than the standard investigation but has the potential to pay off in the longer term in the form of a lower need for downstream investigations, a better quality of life, and fewer cardiac events. Therefore, we plan to follow outcomes and costs for at least 5 years.

### Regional guidelines

In Region Östergötland, CCTA was introduced as the standard investigation modality for ruling out CAD in all primary care centers in June 2021, when the risk of CAD is assessed as low (PTP < 15%) and further investigation is clinically indicated. The modality CCTA is an excellent rule-out test for significant CAD as it has high sensitivity for detection of coronary stenoses defined by findings on invasive coronary angiography, but its significance must be evaluated by functional investigations (11, 35). To evaluate the PTP concept, scoring PTP according to ESC guidelines from 2019 is mandatory on the referral form for CCTA in Region Östergötland (Fig. 4). A systematic analysis of CCTA results and their connection to PTP estimates is in progress.

The echocardiogram may be omitted only in very young patients with a high suspicion of an extra-cardiac cause and in patients with multiple morbidities where the result has no impact on further clinical management (8). In Region Östergötland, the standard investigation recommended in primary health care is still exercise bicycle stress test and consideration of echocardiography when the risk of significant CAD is at least intermediate (PTP > 15%). Given the opportunity to assess the value of echocardiography in the entire trial group, we decided to make it mandatory in the standard investigation group as part of the trial.

A recently published study employed heart sound analyses using an acoustic CadScore device on low-risk patients to reclassify them as having a lower likelihood of disease, rendering further testing unnecessary (36). The CadScore calculation has been tested in populations at different risk levels (37, 38), but the potential to rule out significant coronary stenosis in patients with moderately increased risk derived from a primary care setting has, to the best of our knowledge, not been studied before.

The package investigation includes CAC-scoring, which may assist physicians in assessing the indication for statin (and possibly aspirin) treatment and subsequently may influence patients’ adherence to prescribed medications and potentially reduce the incidence of cardiovascular events over time (39). We will compare the two trial groups and conduct subgroup analyses on prescription and adherence to cardioprotective drugs, if deemed reasonable.

The trial design provides an excellent opportunity to compare the package investigation with the standard investigation. Although the exercise stress test has inferior diagnostic accuracy compared to more advanced functional imaging tests, which has led to their removal from guidelines in the UK as early as in 2010 (11, 35), local regional guidelines continue to recommend the exercise stress test as an initial examination in primary care. This recommendation is based on the long-standing experience of referring physicians, test providers, and cardiologists in the region. Given this recommendation, it was considered ethical to retain the test as the initial screening for patients in primary care at intermediate risk for CAD during the approximately 3-year inclusion period of the trial.

### Trial status

The trial was opened for recruitment of participants on the 1 st of May 2023. The recruitment process is planned to be completed approximately on the 30th of April 2026. The actual protocol version is 1.1 date 31-Jan-2025.

## Data Availability

The steering committee has access to the final trial dataset.

## Abbreviations

AE: Adverse event
CABG: Coronary artery bypass grafting
CAC: Coronary artery calcification
CAD: Coronary artery disease
COPD: Chronic obstructive pulmonary disease
CCTA: Coronary computed tomography angiography
CMIV: Centre for Medical Imaging Science and Visualization
CT: Computed tomography
ECG: Electrocardiogram
ESC: European Society of Cardiology
MACE: Major adverse cardiovascular events
MRI: Magnetic resonance imaging
NSTEMI: Non-ST elevation myocardial Infarction
PCI: Percutaneous coronary intervention
PHC: Primary Healthcare Center
PTP: Pre-test probability
PI: Principal investigator
SAE: Serious adverse event
SPECT: Single-photon emission computed tomography
STEMI: ST elevation myocardial infarction
